# Distinct Evolutionary Profiles and Functions of microRNA156 and microRNA529 in Land Plants

**DOI:** 10.3390/ijms222011100

**Published:** 2021-10-14

**Authors:** Qi Xie, Xufeng Wang, Juan He, Ting Lan, Jiayu Zheng, Yupeng Li, Jinkang Pan, Ling Lin, Junyi Zhao, Jing Li, Yu Yu, Beixin Mo, Xuemei Chen, Lei Gao, Lin Liu

**Affiliations:** 1 Guangdong Provincial Key Laboratory for Plant Epigenetics, Longhua Bioindustry and Innovation Research Institute, College of Life Sciences and Oceanography, Shenzhen University, Shenzhen 518060, China; xie11185566@szu.edu.cn (Q.X.); xufengw@ucr.edu (X.W.); lanting@szu.edu.cn (T.L.); 2019302023@email.szu.edu.cn (J.Z.); liyupeng2019@email.szu.edu.cn (Y.L.); 1900251028@email.szu.edu.cn (J.P.); linling2017@email.szu.edu.cn (L.L.); junyizhao@szu.edu.cn (J.Z.); lijing8311@szu.edu.cn (J.L.); yuy@szu.edu.cn (Y.Y.); bmo@szu.edu.cn (B.M.); 2 Key Laboratory of Optoelectronic Devices and Systems of Ministry of Education and Guangdong Province, College of Optoelectronic Engineering, Shenzhen University, Shenzhen 518060, China; 3 Life Sciences and Medicine, University of Science and Technology of China, Hefei 230026, China; hejuan2018@ustc.edu.cn; 4 Department of Botany and Plant Sciences, Institute of Integrative Genome Biology, University of California, Riverside, CA 92521, USA; xuemei.chen@ucr.edu

**Keywords:** miR156, miR529, *SQUAMOSA promoter binding protein-like* genes, evolution, land plants

## Abstract

MicroRNA156 (miR156) and miR529 have high sequence similarity and recognize overlapping sites in the same target genes, *SQUAMOSA promoter binding protein-like* (*SPL* or *SBP* box) genes, making it difficult to accurately distinguish their roles in regulatory networks that affect numerous biological functions. Here, we collected data about miR156 and miR529 family members from representative land plants and performed sequence comparisons, phylogenetic analysis, small RNA sequencing, and parallel analysis of RNA ends (PARE) analysis to dissect their evolutionary and functional differences. Although miR156 and miR529 are highly similar, there are differences in their mismatch-sensitive regions, which are essential for target recognition. In land plants, miR156 precursors are conserved mainly within the hairpin region, whereas miR529 precursors are conserved outside the hairpin region, including both the 5’ and 3’ arms. Phylogenetic analysis showed that *MIR156* and *MIR529* evolved independently, through divergent evolutionary patterns. The two genes also exhibit different expression patterns, with *MIR529* preferentially expressed in reproductive tissues and *MIR156* in other tissues. PARE analysis revealed that miR156 and miR529 possess specific targets in addition to common targets in maize, pointing to functional differences between them. Based on our findings, we developed a method for the rapid identification of miR529 and miR156 family members and uncovered the evolutionary divergence of these families, providing insights into their different regulatory roles in plant growth and development.

## 1. Introduction

MicroRNAs (miRNAs) are 21–24 nucleotide (nt) long noncoding RNAs that regulate many physiological and developmental processes in plants and animals [1]. In the past two decades, extensive studies have uncovered the evolution, metabolism, and functions of miRNAs [2,3]. In plants, each miRNA gene is transcribed by RNA polymerase II, thereby producing a pri-miRNA, which is then cleaved by dicer-like 1 (DCL1) and its accessory proteins serrate (SE) and hyponastic leaves (HYL1) [4]. This process leads to the formation of pre-miRNA followed by the release of the miRNA-5p/miRNA-3p duplex [4,5]. The miRNA strand is selectively loaded into an argonaute (AGO) protein (generally AGO1) to form an RNA-induced silencing complex (RISC), which targets specific mRNAs for cleavage or translational repression [4,5,6].

Plant miRNAs recognize nearly complementary sequences that are generally located within the coding sequences of their targets [7]. Thus, when predicting miRNA targets, the following factors should be considered: the degree of miRNA-target complementarity, the conservation of target genes in closely related species, miRNA and target gene expression profiles, the secondary structures of the target sites, and mRNA-binding energies [7,8]. The mismatch-sensitive (MS) region of a plant miRNA is located at nucleotides 2–13 from the 5’ end. This region is essential for miRNA activity, as it undergoes complementary pairing with its target [7,9,10]. The MS region is the most important region to consider when classifying plant miRNAs into gene families [7,10]. The variation in miRNA targets is also important in investigating similar miRNA families, such as the miR159 and miR319 families [11,12].

The sequences of miR156 and miR529 family members are highly similar, and both target *SQUAMOSA promoter binding protein-like* (*SPL* or *SBP* box) genes, which encode transcription factors that share a common SBP domain [13,14]. Many studies have focused on the functions of the highly conserved miR156 family, whereas there have been relatively few studies of miR529 [15,16,17,18]. miR156-*SPL* is a broadly investigated regulatory module in plants. By regulating *SPL* expression, miR156 is involved in regulating many biological processes, including flowering time, branching/tillering, and environmental stress responses [14,15,16,19,20]. Considering the high degree of sequence similarity between miR529 and miR156, it would be interesting to determine whether miR529 functions independently, redundantly, or synergistically with respect to miR156.

miR156 is preferentially expressed in rice seedlings, whereas miR529 is mainly expressed in panicles, and both miRNAs confer complex spatiotemporal regulation of their target gene *ideal plant architecture1* (*IPA1*), which has pleiotropic effects on rice plant architecture [21]. A study exploring the evolutionary different of *SPL* genes targeted by miR156 and miR529 in three plant species (moss (*Physcomitrella patens*), rice (*Oryza sativa*), and maize (*Zea mays*)) revealed that miR529 targets comprise a subset of miR156 targets and that the targets regulated by miR156 alone and by both miR156 and miR529 are under different levels of selection pressure [22]. A few studies have sought to dissect the differences between the two miRNA families. miR529 precursors, and especially mature miR529 sequences, evolve more rapidly than miR156 precursors, and *MIR529* genes have a higher average loss rate than *MIR156* genes after identical duplication events [23]. Interestingly, miR529 can be detected in bryophyta and monocots [24,25,26,27] but is absent in intermediate species (some lycophytes and coniferophyte) and eudicots [26,27]. Interestingly, despite the loss of miR529s, some specific eudicot *SPL* family genes have retained miR529 target sites [28]. Transgenic *Arabidopsis thaliana* plants overexpressing a miR529 precursor from a monocot that naturally lacks miR529 displayed phenotypes similar to that of an *spl* mutant, indicating that the retained target sites of miR529 were still functional [28]. However, no method is currently available to effectively distinguish certain *MIR529* and *MIR156* genes.

Some annotated pre-miR156s (such as maize Zma-miR156j) also contain a 21-nt sequence that can be annotated as miR529 in the stem region just behind the mature miR156 sequence. Perhaps these annotated pre-miR156s can also generate miR529 by alternative processing. In some cases, different miRNA family members are indeed formed by alternative processing, such as the rice miR444 family members miR444a.1 and miR444a.2 [29] and *A. thaliana* miR161 family members miR161.1 and miR161.2 [30]. Some ancient and highly conserved *MIRNAs* were also found to undergo alternative processing, such as *MIR319C* in melon (*Cucumis melo* L.) [31]. Small RNA sequencing data showed that miR319c precursor could not only generate miR319c but also produce an alternative #miR319c with very different sequences under cold stress [31]. It is important to determine whether the annotated pre-miR156s that include miR529 sequences can generate miR529s. Significant numbers of divergent and convergent gene pairs have been found in many plant species [32] and whether miR156 and miR529 underwent divergent or convergent processes remains elusive. The relationship between miR156 and miR529 required further investigation to better understand their functions in plants.

In the present study, we developed a rapid method to distinguish *MIR529* from *MIR156* based on their sequence features. We also predicted miR156 and miR529 family members from 50 land plants for which genome sequences were available to investigate their evolutionary relationship. *MIR156* is phylogenetically distant from *MIR529*, and they exhibit different expression patterns and possess specific target genes in addition to common ones. This study illustrates the evolutionary differences between miR156 and miR529 and provides insights into their regulatory roles in plants from an evolutionary perspective.

## 2. Results

### 2.1. Sequence Comparison Reveals Differences in the Mismatch-Sensitive (MS) Regions of miR156 and miR529

**Figure 1:**
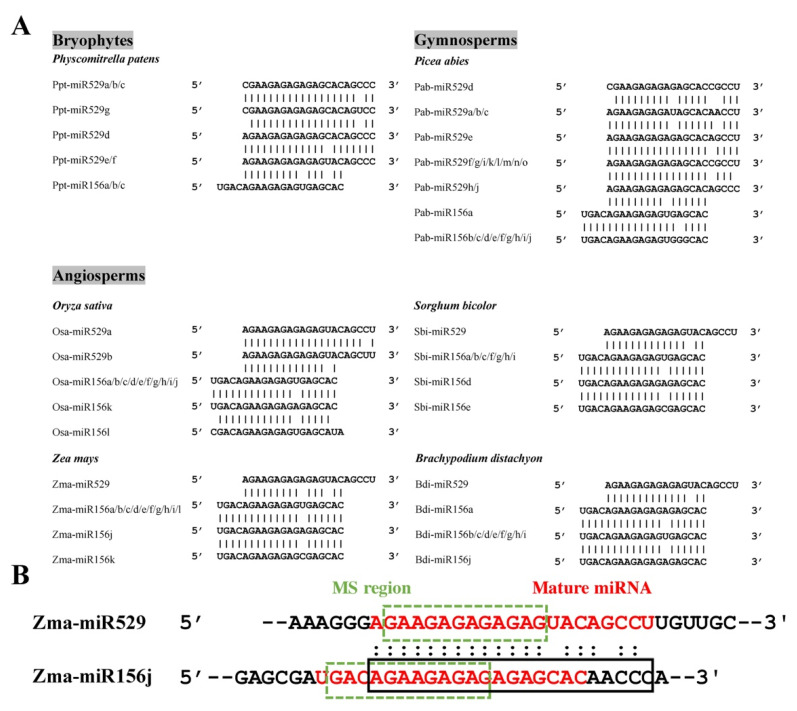
Sequence comparison of miR156 and miR529 in various plant species. (**A**) Multiple sequence alignment of miR529 and miR156 family members in six representative plant species with available sRNA-seq support. (**B**) Sequence comparison of Zma-miR529 and Zma-miR156j. The mature miRNA sequences are indicated in red. The mismatch-sensitive (MS) regions are marked by green dotted rectangles. The miR529-like sequence in the precursor of Zma-miR156j is marked by a black rectangle.

To explore the relationship between miR529 and miR156, we downloaded their precursor and mature sequences from miRbase 22 [33] and selected those supported by small RNA sequencing (sRNA-seq) data from six representative plant species for analysis, including a bryophyte (*P. patens*), a gymnosperm (*Picea abies*), and four angiosperm species (*O. sativa*, *Z. mays*, *B. distachyon*, and *Sorghum bicolor*) [34,35,36,37,38,39,40]. Sequence comparison showed that miR156 and miR529 family members are highly similar, with ~13–16 overlapping nucleotides (Figure 1A, Table S1). For example, Pab-miR529a/b/c share 13 identical nucleotides with Pab-miR156b/c/d/e/f, and Bdi-miR529 and Bdi-miR156a share 16 overlapping nucleotides. Strikingly, the sequences of miR156 family members frequently begin with the four nucleotides UGAC or CGAC at their 5’-ends, representing a characteristic that may distinguish miR156 from miR529 (Figure 1A). More importantly, the last three nucleotides, “GAC”, lie in the MS region, which is critical for the targeting and classification of miRNAs.

Numerous miR156 precursors also contain a 21-nt sequence that can be potentially annotated as miR529. Taking Zma-miR156 as an example, a sequence (shown in the black rectangle) with only a 3-nt difference from Zma-miR529 was found in the Zma-miR156j precursor just behind the mature miR156 sequence (Figure 1B). This observation raises the question of whether the miR529-like sequence can be generated from the annotated miR156 precursor. We searched public sRNA-seq datasets [37,41,42] and did not detect any reads corresponding to a miR529-like sequence, suggesting that the miR529-like sequence in the Zma-miR156 precursor is not actually produced as a miRNA. Similar cases were found for other plant species. Using the miR529 sequence as a query for BLAST analysis against the precursor sequences in miRbase 22, we identified many annotated miR156 precursors containing miR529-like sequences. We retained 22 of these sequences from species with available sRNA-seq data for further analysis. Again, no miR529-like sRNAs were detected (Table S2). In addition to miR529-like sequences in miR156 precursors, we also detected miR156-like sequences in miR529 precursors, including precursors of Aqc-miR529 from columbine (*Aquilegia coerulea*) and Far-miR529 from tall fescue (*Festuca arundinacea*) (Table S2). Since the first four nucleotides of Aqc-miR529 and Far-miR529 are UGAC, a sequence commonly found at the 5’ ends of miR156 family members, it is highly likely that these miRNAs were mistakenly annotated as miR529s in miRbase.

### 2.2. Phylogenetic Analysis Shows That MIR156 and MIR529 Evolved Independently and Exhibit Divergent Evolutionary Patterns

**Figure 2:**
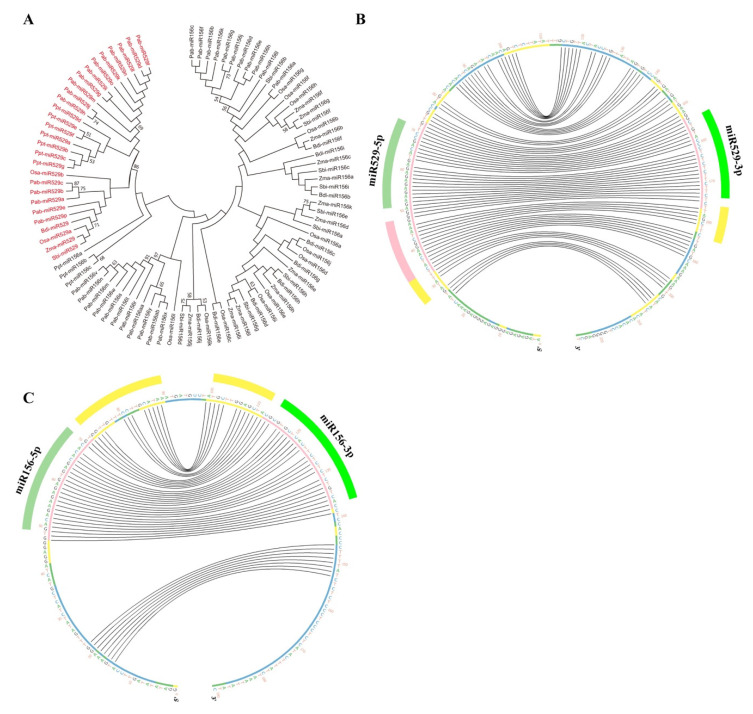
Phylogenetic analysis and sequence alignment of *MIR156* and *MIR529* genes. (**A**) Phylogenetic analysis of miR156-5p/loop/miR156-3p and miR529-5p/loop/miR529-3p in six representative plant species. Multiple sequence alignments of miR156-5p/loop/miR156-3p and miR529-5p/loop/miR529-3p were conducted via MAFFT using Clustal W2, and the phylogenetic tree was constructed by the neighbor-joining method and drawn by Mega 7.0. (**B**,**C**), multiple sequence alignments of miR529 and miR156 family members in representative plant species with available sRNA-seq data. Circos plots of miR529 precursors from six representative species (**B**) and miR156 precursors from four representative angiosperm species (**C**) are shown. The outermost dark-green and light-green bars denote miRNA-5p and miRNA-3p, respectively. The outermost pink and yellow bars denote the conserved regions (yellow: 40–70% conservation, pink: >70% conservation). Different colors inside the nucleotide sequences represent the degree of sequence similarity (blue: <20%; green: <40%; yellow: 40–70%; pink: >70%). Black curved lines connect bases that interact with each other in the secondary structures of the precursors.

We collected pre-miR529 and pre-miR156 sequences provided by miRbase 22 from the six plant species mentioned above with available genome sequences and sRNA-seq support (Table S3). In *P. abies*, the mature sequences of Pab-miR156i, Pab-miR156p, Pab-miR156u, and Pab-miR156q lie in the 3’ end of the precursor sequence. This pattern differs from that of most mature miR156 sequences, which commonly lie in the 5’ arm, so we omitted these four precursors from further analysis. In most miRNA precursors, the sequences from miRNA-5p to miRNA-3p are highly conserved. Thus, we used this sequence, which we named miRNA-5p/loop/miRNA-3p, for phylogenetic analysis. miR529-5p/loop/miR529-3p and miR156-5p/miR156-3p were divided into two distinct clades with strong bootstrap support (Figure 2A).

The conservation of plant *MIRNAs* extends beyond the miRNA-5p/loop/miRNA-3p region. These conserved regions correspond to structural determinants that are recognized during the biogenesis of plant miRNAs [27]. To evaluate whether *MIR156* and *MIR529* show differences in sequence conservation, we downloaded their corresponding miRNA-5p/loop/miRNA-3p sequences, along with 40 bp adjacent upstream and downstream sequences (40 bp-miR156-5p/loop/miR156-3p-40 bp and 40 bp-miR529-5p/loop/miR529-3p-40 bp), using Phytozome v.12.1 (Table S3) and performed multiple sequence alignment of the six representative plant species with T-Coffee [43,44]. The regions and degrees of conservation between 40 bp-miR529-5p/loop/miR529-3p–40 bp sequences were quite different from those of 40 bp-miR156-5p/loop/miR156-3p-40 bp sequences (Figure 2B,C, and Figure S1). While both were conserved in the miRNA-5p and miRNA-3p regions, *MIR529* and *MIR156* had different additional conserved sequences towards the base and loop, respectively, in the stem-loop precursors, pointing to differences in processing during miR156 and miR529 biogenesis. Approximately 17 nt before miR529-5p were highly conserved (>70%), while approximately 10 nt following miR529-3p were relatively conserved (40–70%). Therefore, the additional conserved sequences of *MIR529* genes are located outside the miR529-5p/loop/miR529-3p region (Figure 2B).

Because there are numerous miR156 family members, we analyzed the sequence conservation of *MIR156* genes in gymnosperm and angiosperm species individually. In *P. abies* (Figure S1A), *O. sativa*, *Z. mays*, *B. distachyon*, and *S. bicolor* (Figure 2C), the additional conserved sequences are located within the miR156-5p/loop/miR156-3p region. We were also interested in the above-mentioned *MIR156s* that contain miR529-like sequences. The miR156-5p/loop/miR156-3p regions of these *MIR156s* were highly conserved, further supporting the notion that they are more likely *MIR156* rather than *MIR529* genes (Figure S1B). The conserved region of a pre-miRNA is associated with the manner in which it is processed [27]. The differences in the conserved regions between *MIR529* and *MIR156* indicate that these pre-miRNAs are processed differently: miR156 might be processed in a loop-to-base direction, while miR529 might be processed in a base-to-loop direction.

### 2.3. The Evolutionary History of miR529 and miR156 in Land Plants

**Figure 3:**
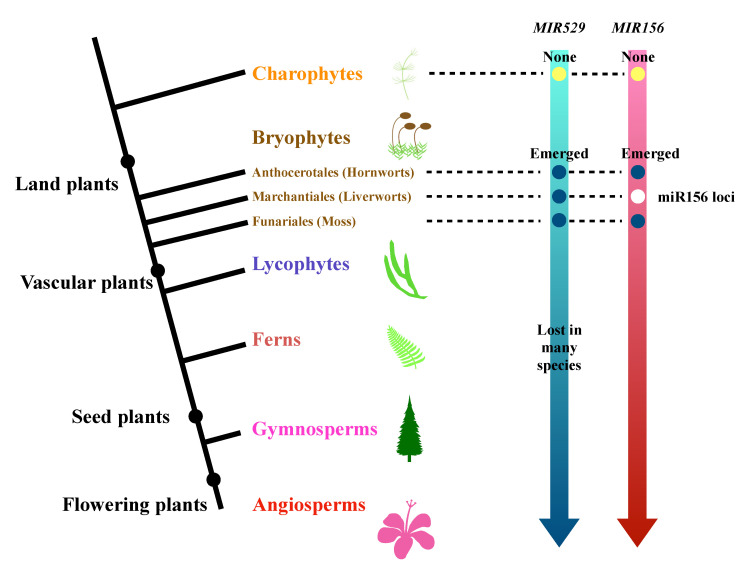
Evolutionary history of miR156 and miR529 in land plants. The evolutionary history of miR156 and miR529 is represented by a simplified phylogenetic tree of the green lineage. Major evolutionary events are labeled on the arrows. “Emerged” marked with a dark blue dot indicates that the *MIR156* or *MIR529* gene was present, encoding the mature miRNA as part of a hairpin precursor, and “miR156 loci” marked with a white dot indicates the presence of the 21-nt miR156 sequence, but not a hairpin precursor, in the genome. In addition, the mature miR156 RNA was not detected in the sRNA-seq datasets.

To explore the evolutionary history of miR529 and miR156, we identified predicted *MIR529* and *MIR156* genes from 56 species (50 plant species and 6 algae species) for which genome sequences were available [45,46,47,48]. miR156 or miR529 was considered to exist in a plant species when at least one genomic locus or transcript was detected that could form a proper hairpin structure. In total, we identified miR529s from 30 plant species and miR156s from 48 species and used them to perform phylogenetic analysis (Figure 3, Figure S2, Table S4). miR156 family members were detected in all land plant species except for two species belonging to Marchantiophyta (liverworts; Figure 3, Figure S2, Table S4). We predicted the secondary structures of 20 bp-miRNA-5p/loop/miRNA-3p-20 bp sequences using RNAfold, as shown in Table S4.

miR529 was discovered in broyophytes and is regarded as the earliest-diverging clade of land plants [45,49]. miR529 is also found in ferns, gymnosperms, and angiosperms but was lost in many plants, including some basal plants (e.g., *Spirodela polyrhiza*, which is considered to be a basal monocot) during evolution. Notably, miR529 is almost completely absent in core eudicots, but it still exists in most monocots. miR529 family members were detected in only a few eudicots: *Nelumbo nucifera* (a basal eudicot belonging to Proteales), *Vitis vinifera*, *Ficus carica*, and *Cannabis sativa*. Only three orders of Lycophytes remain on Earth: Lycopodiales, Selaginellales, and Isoetales [48]. Based on our prediction, miR529 is not present in Selaginellaceae (within Selaginellales), while Lycopodiales and Isoetales lack sufficient genome references for assessment. Many ferns contain miR529 [50].

We also tried to predict miR156s in broyophytes and obtained three candidate sequences in liverwort *Marchantia polymorpha*, but these sequences are not capable of forming stem-loop structures (Figure S3), and mature miR156 was not detected from the sRNA-seq data [51,52]. Interestingly, the miR156s could be found in hornwort *Anthoceros angustus* (Table S4), which is considered the most basal plant in recent research [24,25].

### 2.4. A New Method to Distinguish miR529 from miR156 among Diverse Plant Species

**Figure 4:**
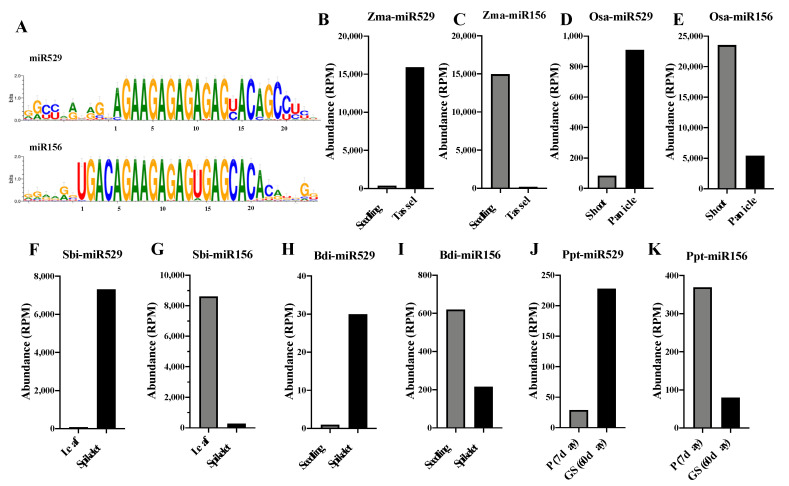
Sequences and expression patterns of miR156 and miR529 family members. (**A**) Sequence logos showing the consensus sequences of miR156 and miR529 family members from land plants. The overall height of each position indicates the conservation at this position (in bits). The height of each nucleotide shows the relative frequency of this nucleotide at this position. (**B**–**K**), expression patterns of miR156 and miR529 in vegetative and reproductive tissues of *Z. mays*, *O. sativa, S. bicolor, P. patens*, and *B. distachyon*, as determined by sRNA-seq. The NCBI GEO IDs of the sRNA-seq datasets are listed in Table S5. P (7 day): protonemata from 7-day-old *P. patens*; GS (60 day): mature gametophores and sporophytes from 60-day-old *P. patens.* RPM represents reads per million.

Our sequence comparison analysis of the six representative plant species revealed that the sequences of miR156 family members begin with the four nucleotides UGAC or CGAC at their 5’ ends at high frequency. To investigate whether this is a common feature in all miR156 family members, we collected data about predicted and experimentally validated miR156 and miR529 family members from 50 plant species for analysis. No UGAC/CGAC motif was detected at the 5’ ends of miR529s, whereas this motif is highly conserved in miR156s (Figure 4A). Therefore, this feature applies to all miR156 family members and could serve as a reliable marker to distinguish miR156 from miR529. When predicting whether a sequence belongs to the miR156 or miR529 family, analyzing the motif at the 5’ end of the miRNA could help rapidly distinguish miR156 from miR529: if the first four nucleotides of a miRNA are UGAC/CGAC, the sequence would be miR156; in all other cases, the sequence is miR529.

Based on this feature, we concluded that Far-miR529 and Aqc-miR529 are incorrectly annotated in miRbase. Indeed, RNAfold analysis indicated that Far-miR529 does not lie in a stem-loop structure, suggesting that it is not a true miRNA. A UGAC sequence is present at the 5’ end of Aqc-miR529 (Figure S4A), suggesting that it is a miR156. We further compared the Aqc-*MIR529* gene with various *MR156* and *MIR529* genes using T-coffee. Aqc-miR529 is more likely to be a miR156 rather than a miR529, as the internal region between Aqc-miR529-5p and Aqc-miR529-3p is similar to those of pre-miR156s (Figure S4B,C), while the outer region (5’ to Aqc-miR529-5p and 3’ to Aqc-miR529-3p) is very different from those of other miR529s (Figure S4D). In addition, we performed phylogenetic analysis of Aqc-miR529-5p/loop/miR529-3p with miR529-5p/loop/miR529-3p and miR156-5p/loop/miR156-3p from the four representative angiosperm plant species, finding that Aqc-*MIR529* is more similar to *MIR156* than to *MIR529* genes (Figure S4E).

### 2.5. Comparative Analysis of the Expression Patterns of miR156 and miR529 in Land Plants

miR156 functions as a master regulator of the juvenile phase in *A. thaliana* by repressing the expression of *SPL* genes; its expression progressively decreases, while the expression of the *SPL* genes increases, as the plant ages and undergoes the juvenile-to-adult transition [53]. In rice, miR156 and miR529 fine-tune the expression of *SPL* genes, thereby affecting many biological functions at different developmental stages. *SPL* genes are repressed by miR156 at the vegetative stage to regulate branch outgrowth and repressed by miR529 at the reproductive stage to define panicle size and architecture [14].

To examine the functional relationship between miR156 and miR529, we used the model plant maize as an example to study the expression patterns of these miRNAs by analyzing sRNA-seq data [37] from vegetative tissue (seedlings) and reproductive tissue (tassels). miR529 was highly expressed in tassels, while miR156s were mainly expressed during vegetative growth (Figure 4B,C). We then investigated the expression patterns of miR156 and miR529 in four other plant species for which there was sRNA-seq data available, including one moss (*P. patens*) and three monocots (*O. sativa*, *B. distachyon*, and *S. bicolor*). The National Center for Biotechnology Information Gene Expression Omnibus (NCBI GEO) identifiers of the sRNA-seq datasets are listed in Table S5. Similar miR156 and miR529 expression patterns were observed in these plants: miR529s are mainly expressed in reproductive tissues, while miR156s are mainly expressed in vegetative tissues (Figure 4D–K). These results reveal distinct expression patterns between miR529 and miR156, indicating that the two miRNAs underwent functional diversification during evolution.

### 2.6. Common and Specific Target Genes of miR156 and miR529 in Land Plants

**Figure 5:**
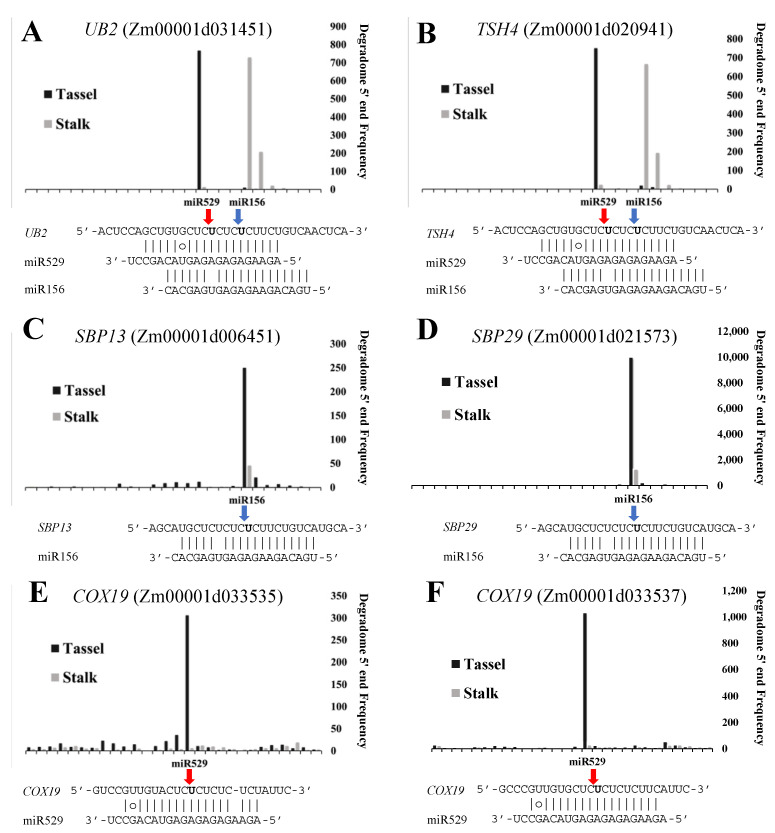
T-plots of miR156 and miR529 target genes in maize. The targets of miR156 and/or miR529 were identified by PARE. Blue and red arrows represent the cleavage sites of miR156 or miR529; histograms represent the abundance of cleavage; *x*-axis represents the nucleotides near the cleavage sites in genomic DNA of the target genes. (**A**,**B**), T-plots for common targets of miR156 and miR529. (**C**,**D**), T-plots for miR156-specific targets. (**E**,**F**), T-plots for miR529-specific targets.

We constructed PARE libraries to identify the target genes of miR156 and miR529 in maize in order to compare the regulatory activities of the two miRNA families. *SPL* genes are common targets of both miR156 and miR529. Using PARE analysis, we detected a series of *SPL* transcripts cleaved by both miR156 and miR529. The cleavage site of miR529 in the target mRNA is located 4 nt before the cleavage site of miR156. Based on the nucleotide counts at the cleavage site, we generated target plots (T-plots) to illustrate the cleavage status of each target gene (Figure 5 and Figure S5). The T-plots of two representative *SPL* genes, *UB2* and *TSH4*, indicate that these two genes are cleaved by both miR156 and miR529, with a high frequency of cleavage by miR156 in shoots and by miR529 in tassels (Figure 5A,B), which is consistent with the tissue-specific expression patterns of miR156 and miR529.

In addition to common targets, we identified a few miR156- and miR529-specific targets. T-plot analysis showed that two *SPL* genes, *SBP13* (Zm00001d006451) and *SBP29* (Zm00001d021573), are cleaved only by miR156 (Figure 5C,D). miR156 shows a high cleavage efficiency at miR156-specific targets but a low efficiency at common targets. Perhaps a competitive relationship exists between miR156 and miR529 in regulating their common targets. Two miR529-specific target genes, Zm00001d033535 and Zm00001d033537, which belong to the *COX19* family (CHCH domain family) [54], are cleaved by miR529 alone (Figure 5E,F). We looked for orthologs of these miR156- and miR529-specific target genes in other plant species. *OsSPL13* (LOC_Os07g32170) and *BdSBP13* (Bradi1g26720) were identified as miR156 targets in *O. sativa* and *B. distachyon,* respectively (Table S6). *COX19* orthologs were identified in *O. sativa* (Os03g068550) and *B. distachyon* (Bradi1g12230), but no miR529 cleavage site was identified in these genes, suggesting that these genes might not be targeted by miR529 and that *COX19* might be a maize-specific miR529 target.

*SPL* genes in members of the liverwort family are regulated by miR529 but also contain binding sites for miR156 [55]. Although miR156 is not present in *M. polymorpha*, miR156 target sites were detected in the *SPL* genes of this plant. PARE was used to predict miR529 targets in *M. polymorpha* [51], and the results showed that not all miR529 targets were conserved across plant evolution. Many *MYB* family genes are miR529 targets in *M. polymorpha* [51], but these genes are not targets of miR529 in maize or rice (Table S6).

## 3. Discussion

### 3.1. The Distinction between miR529 and miR156

miR529 is an ancient miRNA family that was first reported in rice [34] and whose members were subsequently discovered in many other plant species, such as *S. bicolor*, *P. patens*, and *M. polymorpha* [35,38,52]. Despite more than ten years of research, the biological functions of miR529s remain poorly understood. This is partly attributable to the existence of miR156, a miRNA family with high sequence similarity to miR529. miR156 and miR529 share common targets, i.e., *SPL* genes. Thus, most studies to date have focused on the miR156-*SPL* module, while the miR529-*SPL* regulatory relationship has to some extent been ignored. In the current study, we discovered that miR156 and miR529 exhibit different expression patterns in different tissues and developmental stages, indicating that the miR529-*SPL* module has a unique biological function. It is therefore important to distinguish between these two miRNA families and clarify their evolutionary relationship.

The MS region is an important marker of differentiating miRNAs in plants. Notably, we determined that the MS regions of miR156 and miR529 are not identical: their sequences differ by three nucleotides, GAC, located at positions 2–4. However, this difference cannot be used to distinguish miR529s from miR156s because some pre-miR156s contain miR529-like sequences; thus, we investigated whether such miR156s and miR529s are alternatively processed from a single pre-miRNA. We collected available data for miR156s and miR529s from plant species in which miRNAs were identified by sRNA-seq and found no evidence of miR529-like sequences being produced from pre-miR156s. This finding implies that miR529s are not the result of alternative processing of miR156 precursor sequences. Consistent with this notion, no obvious evolutionary relationship was identified between pre-miR529s and pre-miR156s. Recently, another miRNA-miR535 was found to have sequence similarity to miR156 and miR529, and it has been reported to target *SPL* genes as well [56]. However, the MS regions of miR535s are very different compared to those of miR156s and miR529s, making it easier to distinguish them from miR156s and miR529s [56].

By analyzing the 40 bp-miR529-5p/loop/miR529-3p-40 bp sequences, we found that UGAC/CGAC, the first four nucleotides of miR156, never appear in miR529s, and therefore, this characteristic can be used to rapidly distinguish miR156s from miR529s. The first four nucleotides of Aqc-miR529 in miRbase are UGAC, and the Aqc-*MIR529* sequence has greater similarity to *MIR156* than to *MIR529* genes, suggesting that it was mistakenly annotated as miR529 in miRbase.

### 3.2. Comparative Analysis of the Origins and Continuity of miR156 and miR529 in Land Plants

Our analyses showed that miR529 has frequently been lost in plant species during evolution. We traced the origin of miR529 to early land plants before the emergence of *M. polymorpha*, one of the most ancient land plants. Among green algae, we were not able to determine whether miR529 is present in Zygnematophyta and Coleochaetophyta, which are considered sister groups to land plants, due to the lack of reference genomes for these clades. However, miR529 is absent in other green algae, including *Chlamydomonas reinhardtii*, *Dunaliella salina*, *Volvox carteri*, *Coccomyxa subellipsoidea*, *Micromonas* sp. RCC299, and *Ostreococcus lucimarinus*. We could not determine with certainty whether miR529 appeared in green algae, but we suspect that miR529 was important in the early history of land plants.

### 3.3. miR156 and miR529 Exhibit Different Expression Patterns and Function Cooperatively or Independently by Regulating Common or Specific Targets

The high degree of similarity between miR156 and miR529 raises the issue of whether they participate in common or diversified regulatory pathways. We detected different expression patterns for miR529 compared to miR156 in maize. miR529 levels are high in tassels and relatively low in other tissues, while miR156 is mainly present during the vegetative stage. As *SPL* genes are common targets of miR156 and miR529, they could be regulated by the two miRNAs during different developmental stages or in different tissues. Indeed, some *SPL* genes are targeted by miR156 in shoots and miR529 in tassels.

However, not all *SPL* genes are targeted by both miR156 and miR529: some are miR156-specific or miR529-specific targets. Some *SPL* genes (*SPL3*, *SPL4*, *SPL11*, *SPL12*, *SPL13*) in rice are thought to contain only miR156 target sites [57]. The *SPL13* orthologs Zm00001d006451 and Zm00001d021573 in maize are cleaved only by miR156, as revealed in our PARE analysis. We also detected miR529-specific targets in maize, but these targets are not conserved in rice. PARE analysis revealed that *MYB* genes are targets of miR529 in *M. polymorpha* [51] but are not targeted by miR529 in other plant species. These results suggest that during evolution, in addition to the conserved miR156/miR529-*SPL* module, the two miRNAs might have evolved specific targets and undergone functional diversification to better coordinate plant growth and development.

## 4. Materials and Methods

### 4.1. Prediction of miR156 and miR529 Family Members in Land Plants

Mature sequences of miR156 and miR529 were retrieved from miRBase 22 [33]. To identify miR529 and miR156 family genes from plant species that have not been annotated, mature sequences of known miR529s (Ppt-miR529a-f from *P. patens*, Osa-miR529a-b from rice) and miR156s (Ath-miR156a-h from *A. thaliana*, Ppt-miR156a-c from *P. patens*, Osa-miR156a-l from rice) were used as queries to the search genome sequences by BLAST analysis in the Phytozome v.12.1 (https://phytozome-next.jgi.doe.gov/, accessed on 15 August 2021), FernBase (https://www.fernbase.org/, accessed on 15 August 2021), ConGenIE (https://congenie.org/blast, accessed on 15 August 2021), and NCBI databases (https://blast.ncbi.nlm.nih.gov/Blast.cgi, accessed on 15 August 2021) [43,58,59,60]. The secondary structures of each sequence with hits in the databases, as well as the 250-bp upstream and downstream flanking genomic sequences, were predicted using RNAFold [61]: if the structure formed a hairpin and the hit-sequence was located in the duplex region of the hairpin structure, this sequence was considered to be a miR156 or miR529 candidate. The 250-bp flanking genomic sequences of the candidates were further analyzed by BLAST analysis against miRbase, and hits were classified as miR156 or miR529 family members based on their similarities to pre-miR529 or pre-miR156 sequences.

### 4.2. Prediction of miRNA Targets

The psRNATarget server [8] (https://www.zhaolab.org/psRNATarget/, accessed on 15 August 2021) was used to predict putative targets of miRNAs as follows (last accessed on 15 August 2021). (a) Target prediction: the miRNA was submitted to the “submit small RNAs” section and “cDNA library” was selected with default parameters; (b) cleavage site prediction: both the miRNA and target mRNA were submitted to the “submit small RNAs and targets” section with default parameters. The mismatch between the miRNA and target was required to be less than 3 nt at the 5’ end and less than 6 nt at the 3’ end of the miRNA [10].

### 4.3. RNA Extraction and Library Preparation

Maize cultivar ‘B73’ were planted in a green chamber at 25 °C with 65% relative humidity under a 14-h/10-h light/dark cycle. The stalks from maize seedlings and tassels from maize at R1 stage were collected as samples. Total RNA was extracted from *Z. mays* tissues using TRIzol reagent (Invitrogen, Carlsbad, CA, USA) according to the manufacturer’s instructions. A NanoDrop 2000 spectrophotometer (Thermo Fisher Scientific, Waltham, MA, USA) was used to determine the quality of the extracted RNA, and only RNA of high integrity was used for subsequent analysis.

Parallel analyses of RNA end (PARE) libraries (GEO access: GSE175630) were constructed as previously described [62] and sequenced on the Illumina HiSeq 2500 platform to produce 50 bp single-end reads.

### 4.4. sRNA-Seq Data Processing

Adapter-free sequences of sRNA-seq data (Table S5) were downloaded from the National Center for Biotechnology Information Gene Expression Omnibus (NCBI GEO). To quantify miRNA levels, adapter-free reads were counted and assigned to each miRNA (miRbase 22), with a 1 nt shift being allowed on the two ends. Reads per million mapped reads (RPM) values were calculated for each window. Small RNA (sRNA) abundance was compared between different treatments using the R package DESeq2 [63].

### 4.5. PARE Analysis

Raw sequences were filtered to remove low-quality reads, and adapter sequences (TGGAATTCTCGGG) were removed using an in-house perl script. The remaining sequences were analyzed using CleaveLand 4.3 [64] with -n maize gene sequences, -u maize mature miRNA sequences, -e trimmed PARE sequences, and -t 1. Here, maize gene sequences were downloaded from gramene (release 53); maize mature miRNA sequences were downloaded from miRbase 22. The target plot (T-plot) was generated using CleaveLand results. Candidate target genes were selected based on the criteria *E*-value < 0.05 and cleavage category = 0 (T-plot).

### 4.6. Identification of Homologous Genes

The protein sequences of the miR156 and miR529 target genes were downloaded from MaizeGDB 2018 [65] and used as queries for BLAST analysis against the Phytozome v.12.1 database [43] using default parameters to predict homologous proteins in two monocots, *O. sativa* and *Brachypodium* *distachyon*. The results were analyzed and corrected by manual inspection, and only the longest isoforms were retained as representative protein sequences for each identified homologous gene.

### 4.7. Multiple Sequence Alignment and Visualization

Multiple sequence alignments of 40 bp-miR156-5p/loop/miR156-3p-40 bp (the genomic sequences from 40 bp upstream of miR156-5p to 40 bp downstream of miR156-3p) and 40-bp-miR529-5p/loop/miR529-3p-40 bp (the genomic sequences from 40-bp upstream of miR529-5p to 40-bp downstream of miR529-3p) sequences from different plant species were performed using the command-line version of T-Coffee (Centre for Genomic Regulation, Barcelona, Spain) (http://tcoffee.crg.cat/apps/tcoffee/do:regular, accessed on 15 August 2021) [44]. Mafft_msa (Multiple Methods) and slow_pair (Pairwise Methods) were used to align the sequences. The results are shown as circos plots [27,58]. The level of conservation at each position in the consensus region of the multiple sequence alignment was determined based on the output from T-Coffee. We omitted gaps and used the nucleotides within the 40 bp-miRNA-5p/loop/miRNA-3p-40 bp region to draw circos plots. The black lines in the circos plots show the interactions of base pairs in 40 bp-miRNA-5p/loop/miRNA-3p-40 bp.

### 4.8. Construction of Phylogenetic Trees

miR529-5p/loop/miR529-3p and miR156-5p/loop/miR156-3p were aligned using the MAFFT method with Clustal W2 online software (EMBL-EBI, Hinxton, Cambridgeshire, UK)(https://www.ebi.ac.uk/Tools/msa/mafft/, accessed on 15 August 2021) with default settings, the gap opening penalty is 1.53 and the gap extension penalty is 0.123. Phylogenetic reconstruction was performed by the neighbor-joining (NJ) method with the default model, and phylogenetic trees with 1000 bootstrap replicates were drawn by Mega 7.0 [66].

## Supplementary Materials

The following are available online at www.mdpi.com/article/10.3390/ijms222011100/s1. Supplementary Figure S1. Circos representation of precursors of Pab-miR156s and miR529-like miR156s. Supplementary Figure S2. Prediction of miR529 and miR156 in land plants. Supplementary Figure S3. Predicted miR156 family members in Marchantia polymorpha. Supplementary Figure S4. Comparison between Aqc-miR529 and known miR156s and miR529s. Supplementary Figure S5. Target plots of miR156 and miR529 target genes in maize. Supplementary Table S1. Sequences of mature miR156 and miR529 from six representative plants (P. Patens, P. abies, B. distachyon, O. sativa, S. bicolor and Z. mays). Supplementary Table S2. List of miR156 precursors containing miR529-like sequences. Supplementary Table S3. Sequences of miR156 and miR529 precursors in six representative plants (P. Patens, P. abies, B. distachyon, O. sativa, S. bicolor and Z. mays). Supplementary Table S4. Prediction of miR529 and miR156 in land plants. Supplementary Table S5. NCBI accession numbers of sRNA-seq data. Supplementary Table S6. Homologs of miR156- or miR529-specific targets in B. distachyon, O. sativa, Z. mays and M. polymorpha.
