# Supplementary material for: Distinct Evolutionary Profiles and Functions of microRNA156 and microRNA529 in Land Plants
Source: Int J Mol Sci. 2021 Oct 14;22(20):11100. doi: 10.3390/ijms222011100 (PMC8541648; doi:10.3390/ijms222011100)
Supplement: Supplementary file 1 [file ijms-22-11100-s001.zip › Supplemental/Supplemental Fig/Fig S3.pdf]

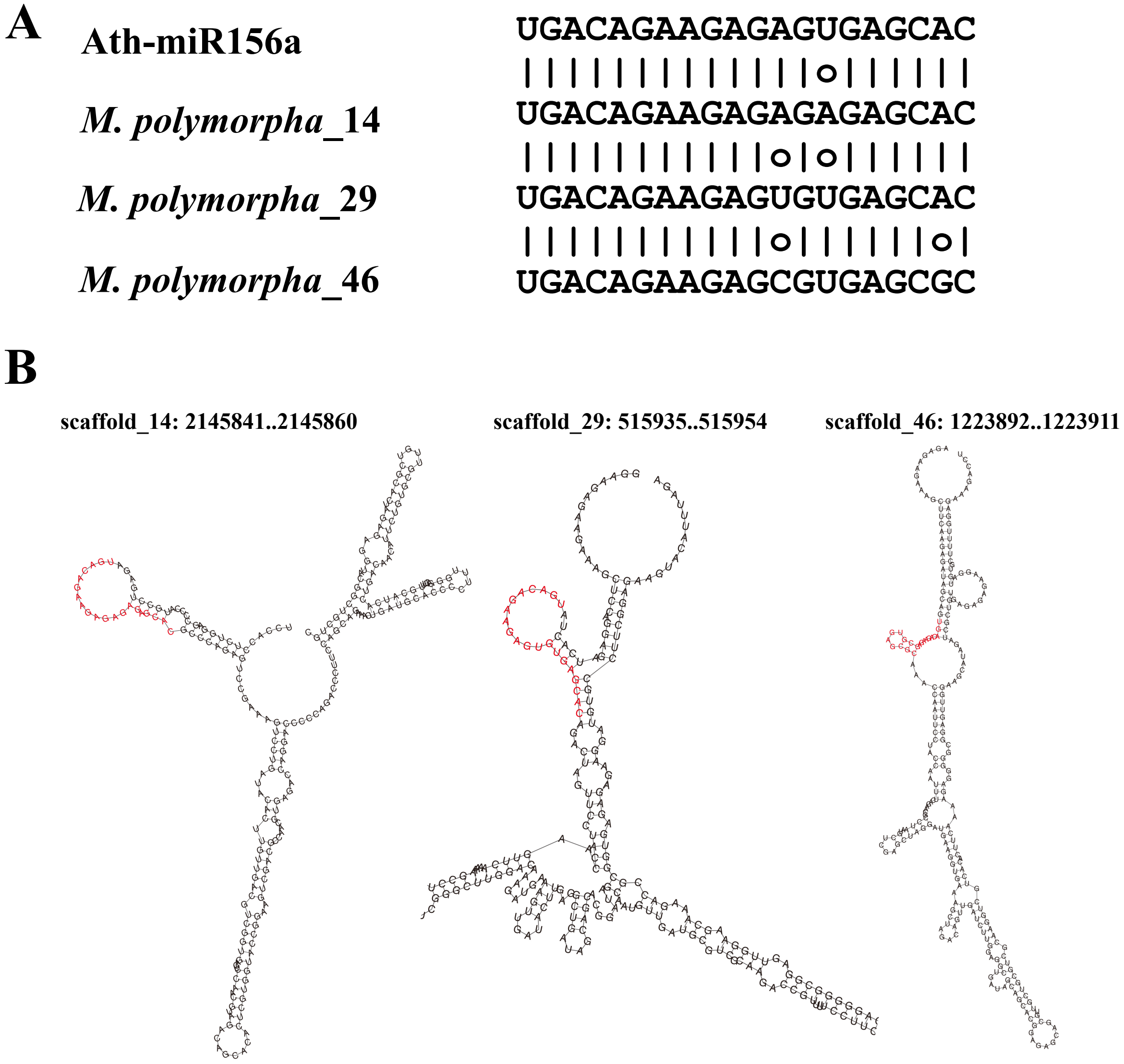
A
Ath-miR156a
UGACAGAAGAGAGUGAGCAC
M. polymorpha_14
UGACAGAAGAGAGAGAGCAC
M. polymorpha_29
UGACAGAAGAGUGUGAGCAC
M. polymorpha_46
UGACAGAAGAGCGUGAGCGC
B
scaffold_14: 2145841..2145860
scaffold_29: 515935..515954
scaffold_46: 1223892..1223911
