# Supplementary material for: Distinct Evolutionary Profiles and Functions of microRNA156 and microRNA529 in Land Plants
Source: Int J Mol Sci. 2021 Oct 14;22(20):11100. doi: 10.3390/ijms222011100 (PMC8541648; doi:10.3390/ijms222011100)
Supplement: Supplementary file 1 [file ijms-22-11100-s001.zip › Supplemental/Supplemental Fig/Fig S1.pdf]

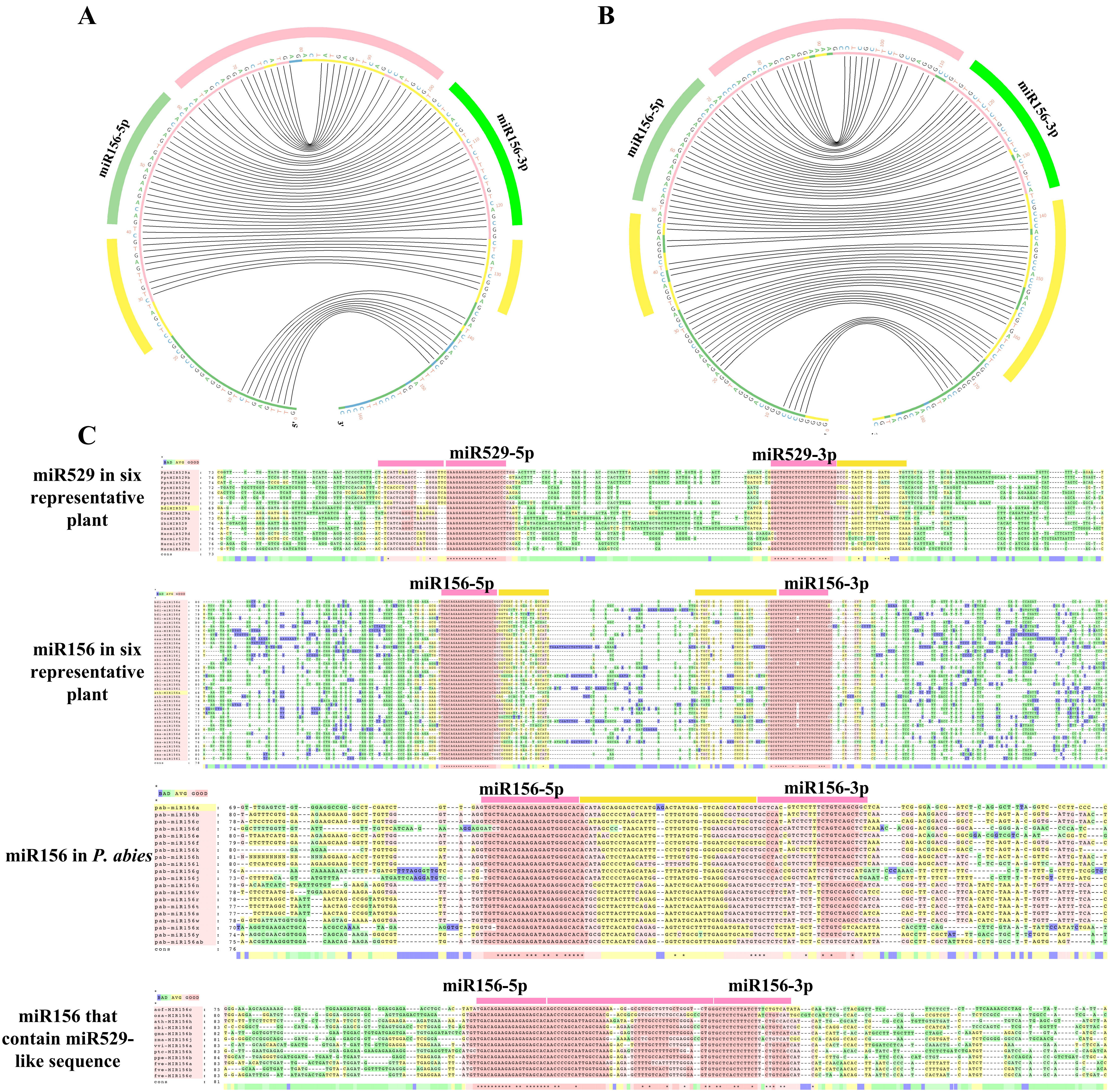
A
miR156-5p
miR156-3p
B
miR156-5p
miR156-3p
C
miR529-5p
miR529-3p
miR529 in six representative plant
miR156-5p
miR156-3p
miR156 in six representative plant
miR156-5p
miR156-3p
miR156 in P. abies
miR156-5p
miR156-3p
miR156 that contain miR529-like sequence
