# Supplementary material for: Distinct Evolutionary Profiles and Functions of microRNA156 and microRNA529 in Land Plants
Source: Int J Mol Sci. 2021 Oct 14;22(20):11100. doi: 10.3390/ijms222011100 (PMC8541648; doi:10.3390/ijms222011100)
Supplement: Supplementary file 1 [file ijms-22-11100-s001.zip › Supplemental/Supplemental Fig/Fig S4.pdf]

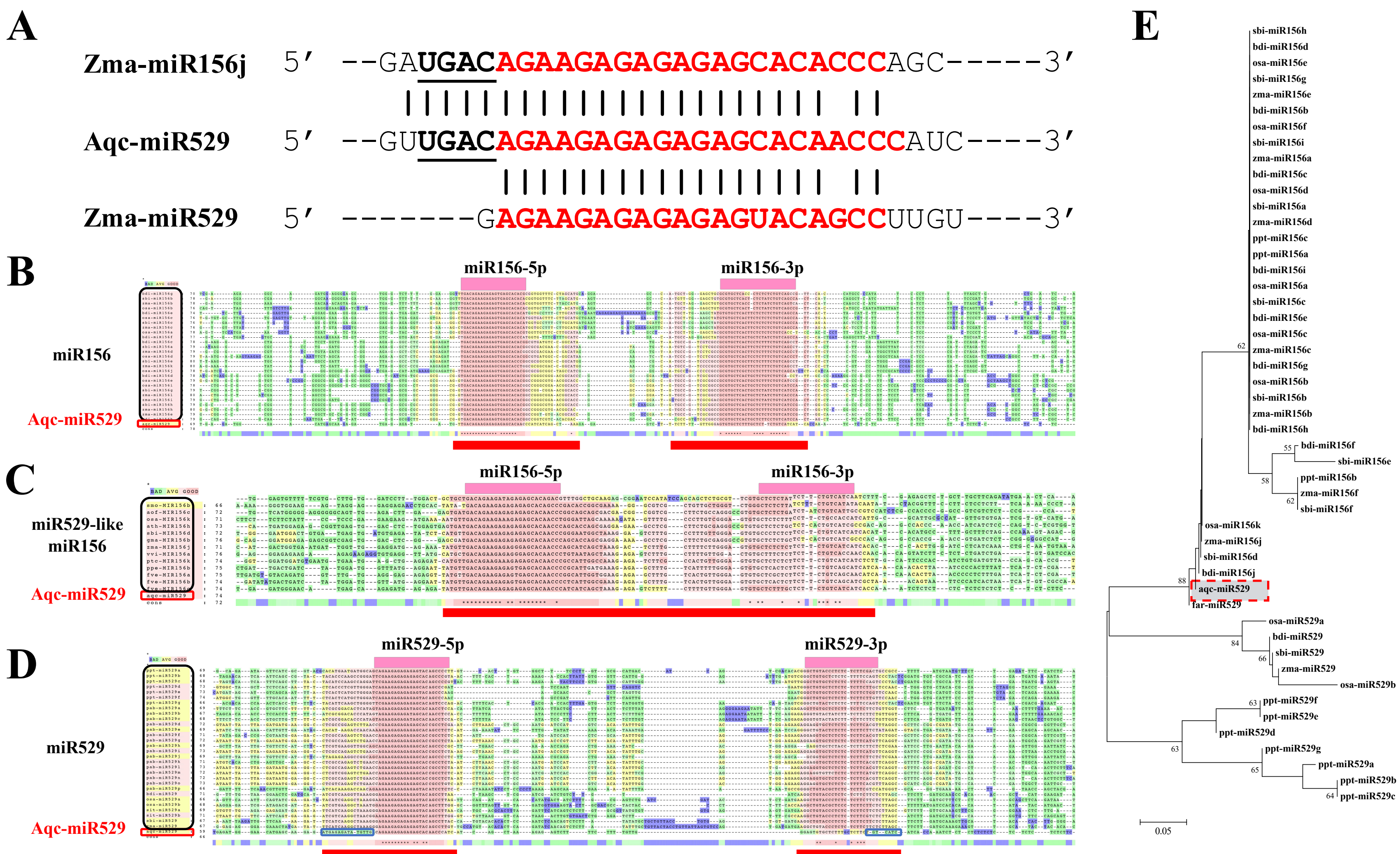
A
Zma-miR156j 5′ --GAUGACAGAAGAGAGAGAGCACACCCAGC-----3′
Aqc-miR529 5′ --GUUGACAGAAGAGAGAGAGCACAACCCAUC----3′
Zma-miR529 5′ -------GAGAAGAGAGAGAGUACAGCCUUGU----3′
B
miR156-5p
miR156-3p
miR156
Aqc-miR529
C
miR156-5p
miR156-3p
miR529-like miR156
Aqc-miR529
D
miR529-5p
miR529-3p
miR529
Aqc-miR529
E
sbi-miR156h
bdi-miR156d
osa-miR156e
sbi-miR156g
zma-miR156e
bdi-miR156b
osa-miR156f
sbi-miR156i
zma-miR156a
bdi-miR156c
osa-miR156d
sbi-miR156a
zma-miR156d
ppt-miR156c
ppt-miR156a
bdi-miR156i
osa-miR156a
sbi-miR156c
bdi-miR156e
osa-miR156c
zma-miR156c
bdi-miR156g
osa-miR156b
sbi-miR156b
zma-miR156b
bdi-miR156h
bdi-miR156f
sbi-miR156e
ppt-miR156b
zma-miR156f
sbi-miR156f
osa-miR156k
zma-miR156j
sbi-miR156d
bdi-miR156j
aqc-miR529
far-miR529
osa-miR529a
bdi-miR529
sbi-miR529
zma-miR529
osa-miR529b
ppt-miR529f
ppt-miR529e
ppt-miR529d
ppt-miR529g
ppt-miR529a
ppt-miR529b
ppt-miR529c
0.05
