# Supplementary figures and images for: Distinct Evolutionary Profiles and Functions of microRNA156 and microRNA529 in Land Plants
Source: Int J Mol Sci. 2021 Oct 14;22(20):11100. doi: 10.3390/ijms222011100 (PMC8541648; doi:10.3390/ijms222011100)

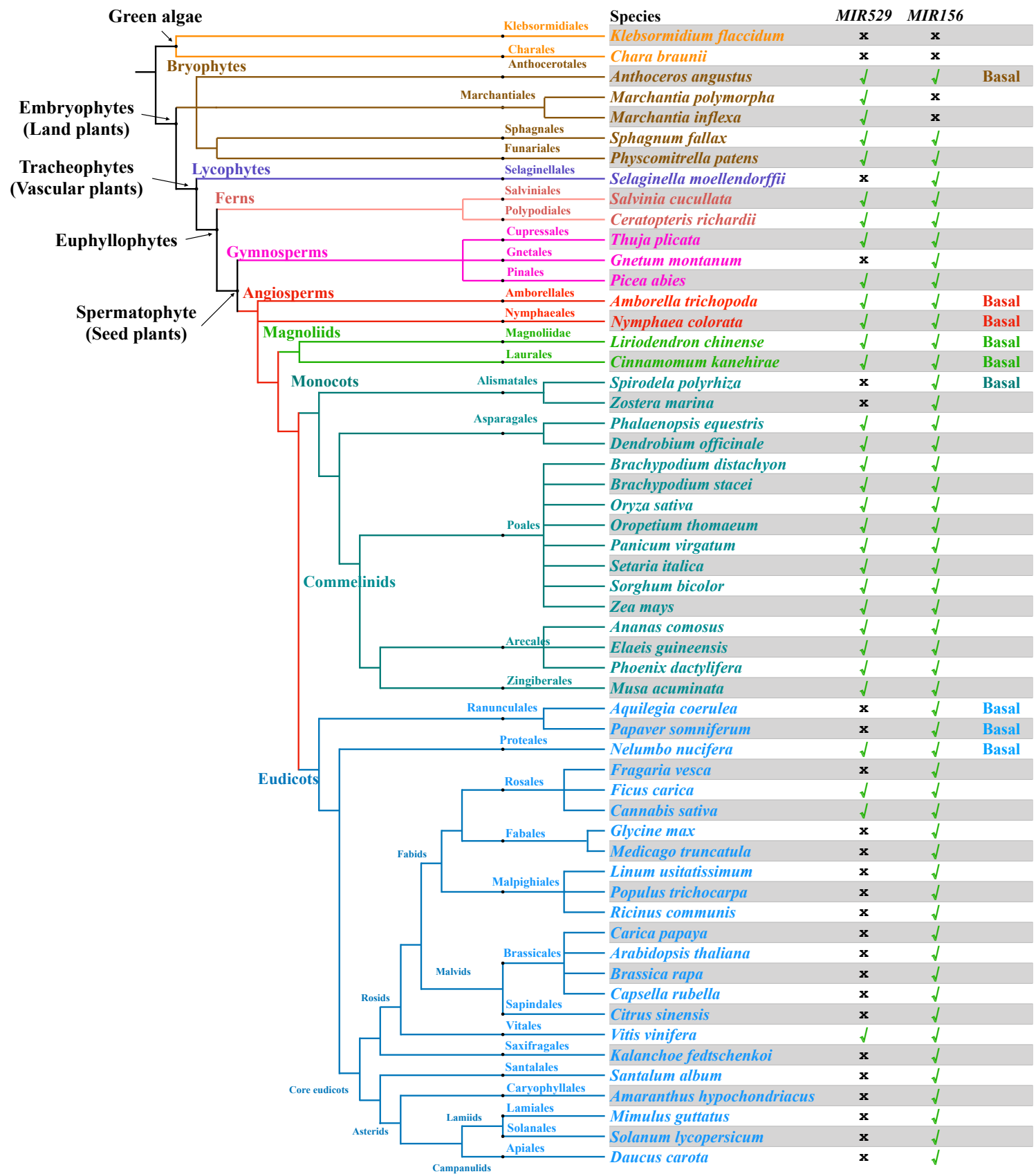

Supplement: Supplementary file 1 [file ijms-22-11100-s001.zip › Supplemental/Supplemental Fig/Fig S2.pdf]

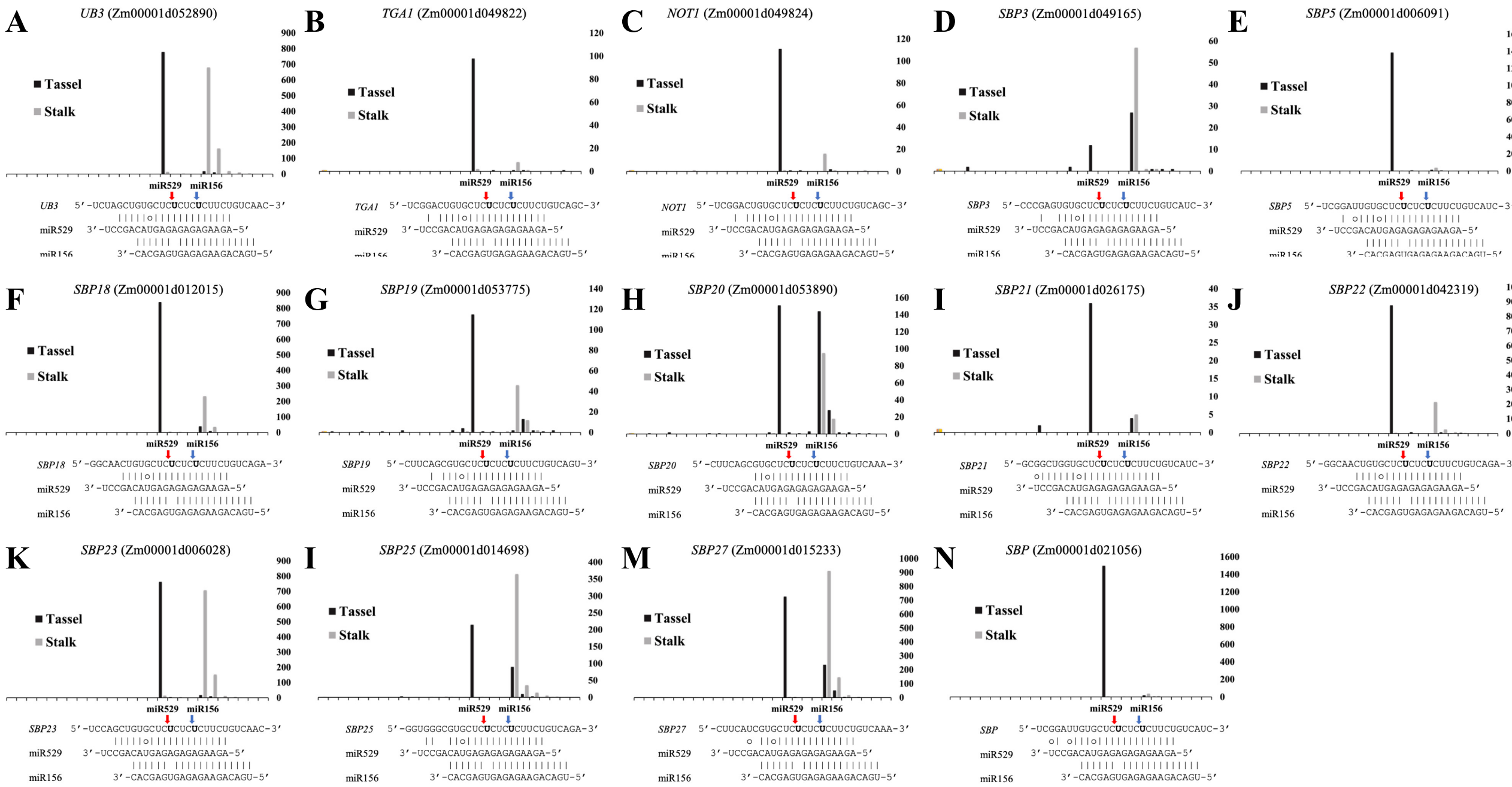

Supplement: Supplementary file 1 [file ijms-22-11100-s001.zip › Supplemental/Supplemental Fig/Fig S5.pdf]
